# Generative artificial intelligence adoption and use in teaching and training healthcare professionals in higher education in the United States: a cross-sectional study

**DOI:** 10.1186/s12909-026-09291-8

**Published:** 2026-04-24

**Authors:** Obinna O. Oleribe, Parichart Sabado, Kazi T. Begum, Matt G. Mutchler, Benedetto Piccoli, Andrew W. Taylor-Robinson, Christopher Denaro, Ricardo Izurieta, Simon D. Taylor-Robinson

**Affiliations:** 1 Department of Health Sciences, School of Public Health and Health Sciences, College of Health, Human Services and Nursing, California State University Dominguez Hills, 1000 E Victoria Street, Carson, CA 90747 USA; 2 Center for Family Health Initiative (CFHI), Orange, CA USA; 3 Center for Computational and Integrative Biology (CCIB), Rutgers University – Camden, Camden, NJ USA; 4 College of Health Sciences, VinUniversity, Hanoi, Vietnam; 5 VinUniversity-University of Illinois Smart Health Center, VinUniversity, Hanoi, Vietnam; 6 Center for Global Health, Perelman School of Medicine, University of Pennsylvania, Philadelphia, PA USA; 7 Department of Surgery and Cancer, Imperial College London, St Mary’s Hospital Campus, London, UK

**Keywords:** Generative Artificial Intelligence, GenAI Training, Healthcare Education, Healthcare Professional, Medical Education, Professional Development

## Abstract

**Background:**

The use of generative artificial intelligence (GenAI) is rapidly expanding across medical and allied health education. However, structured and curriculum-integrated training to prepare future healthcare professionals remains limited. This study examined the adoption, self-reported knowledge, and perceptions of GenAI among faculty and students in a U.S. higher education institution.

**Methods:**

We conducted a cross-sectional study using a self-administered online questionnaire to assess GenAI knowledge, adoption, use, benefits, and challenges among faculty and students in a public Minority Serving Institution in California. Using a convenience sampling technique, data were collected with Qualtrics from January – March 2025 and analysed with IBM SPSS Statistics version 31.

**Results:**

A total of 559 complete responses were analysed (faculty: 19.3%; students: 78.5%). Overall, 83.2% of respondents reported having used GenAI, including 91.1% of faculty and 81.4% of students, with no statistically significant difference by role (*p* = .12). Only 7.9% had received formal training in GenAI, while 29.1% reported having conducted substantial independent research on GenAI (faculty: 48.5%; students: 24.3%). Nearly half of participants (48.7%) perceived GenAI as beneficial for learning, teaching, and research (*p* = .002). Additionally, 47.4% believed GenAI is currently essential, and 70.8% anticipated it will become essential in the future, with significant more faculty respondents asserting the importance of AI (*p* < .001 and *p* = .009, respectively). Most respondents relied on free GenAI tools (90.6%), and among users of paid tools, 66.3% paid out of pocket. Participants primarily sourced GenAI information from the internet (77.3%), reported substantial concerns (70.3%), and experienced multiple challenges in GenAI use (84.7%).

**Conclusions:**

GenAI use among faculty and students is widespread, but formal training and institutional support remain limited. These findings underscore the need for coordinated, campus-wide GenAI training, governance, and policy frameworks to promote ethical, effective, and equitable integration of GenAI into healthcare education and workforce preparation.

**Supplementary Information:**

The online version contains supplementary material available at 10.1186/s12909-026-09291-8.

## Background

Generative artificial intelligence (GenAI) is rapidly transforming healthcare delivery, administration, and education, offering substantial opportunities to improve clinical outcomes, optimize operational efficiency, and personalize learning and care experiences. Across the healthcare continuum, from the education and training of future health professionals to patient management, clinical decision support, and health data analysis, GenAI tools and technologies are reshaping how knowledge is generated, applied, and disseminated. Within higher education, GenAI is increasingly being integrated to enhance student learning, accelerate research workflows, streamline administrative processes, and deliver personalized academic support to faculty and students [1, 2]. As adoption expands, GenAI is redefining how educators, institutional leaders, and learners engage with information, support decision-making, and deliver educational and health-related services, underscoring its growing influence on academic practice and healthcare workforce development [3–8].

GenAI is supporting university students and faculty in (1) analysing individual learning patterns to tailor educational content, ensuring that students receive instruction suited to their unique needs and pace of learning [3]; (2) evaluating assignments and provides immediate, constructive feedback allowing educators to focus more on interactive teaching [4]; (3) scheduling, resource allocation, and monitoring student progress, thereby reducing the administrative burden on educational institutions [5]; (4) providing enhanced support for students with disabilities and multilingual learners [6]; (5) promoting inclusivity in education making learning more engaging while fostering a deeper connection between students and their study materials [7]; and (6) supporting students learning, brainstorming, idea generation, research, feedback, time management, and report development [8–11].

Upon graduation, individuals may work in healthcare settings that leverage GenAI to improve patient outcomes, influence how clinicians accurately interpret images and make diagnoses, enhance workflow and staff scheduling, and reduce medical errors in healthcare systems, while also enabling patients to access and process their data to promote health [1, 12]. Despite the benefits of GenAI, its adoption and use in learning, teaching, and research face multiple challenges. These include hallucinations, transparency, data ownership, and the appropriate and responsible use of GenAI, as well as resistance to its adoption from some faculty and students [13, 14]. Additionally, there are disparities in access to quality GenAI products, concerns about job loss, issues with data privacy and security, data quality and preprocessing, model interpretability, bias in data/algorithms, user interface, acceptability, user engagement, and fear of obsolescence [13–15]. Although several types of GenAI are already in use at various levels, educators and students face challenges in adopting GenAI due to limited access, inadequate technical training, and multiple infrastructure constraints [16–18]. To facilitate AI adoption and use by students post-college, there is a need to introduce them to AI early and educate them on responsible and appropriate use of AI in school and outside college. This is why it is important to investigate its adoption and integration into education.

This study examined levels of GenAI adoption and use, and self-reported knowledge among faculty and students at a large urban minority-serving institution, as current evidence is beginning to highlight the expanding AI divide between the haves and the have-nots and multiple user groups [19]. We intentionally studied an institution with a Hispanic, Black, and Minority-serving status that provided a diverse demographic frequently overlooked in innovation diffusion research to see if they would have widely different perspectives towards GenAI, as well as provide tailored evidence for AI policy development and implementation. By centering our study on this population, we sought to address the systemic underrepresentation of minority-serving environments in the existing literature.

## Methods

### Study design

We conducted an online cross-sectional study in accordance with the Strengthening the Reporting of Observational Studies in Epidemiology (STROBE) guidelines for cross-sectional studies to examine knowledge, adoption, and use of generative artificial intelligence (GenAI) among faculty and students in higher education [20, 21].

### Setting and study population

The study was conducted within a Minority, Black, Hispanic Serving Institution in California. We targeted faculty members and enrolled students. Eligible participants were individuals aged 18 years or older who were actively engaged in teaching, learning, or academic activities during the study period. Participation was voluntary, and no restrictions were placed on academic discipline or level.

California State University, Dominguez Hills (CSUDH) is a minority-serving public institution (MSI) with a 68.2% Hispanic/Latino and 12.2% Black/African American population [22]. Over 80% of the students at CSUDH are from underserved communities, 44.5% are first-generation scholars, and 60.3% are eligible for the US Federal Pell Grant, a program that is designed to assist students from low-income households. In addition, 40.7% are both first generation and underserved, while 52.8% of all students are both underserved and eligible for Pell and other sources of financial aid to support their college attendance.

Participants were drawn from six academic colleges within the institution to reflect a broad cross-section of educational programs. Most respondents were affiliated with the College of Health, Human Services, and Nursing, which houses healthcare and allied health training programs, including disciplines directly involved in clinical practice, public health, and health services delivery. The remaining participants were distributed across non-health colleges, providing contextual breadth while preserving the primary healthcare orientation of the study population. College-level affiliation was used for descriptive purposes only to contextualize the sample and was not employed as an analytic variable to avoid divisive comparisons and maintain institutional neutrality.

### Sample size determination and sampling strategy

Sample size was calculated using an assumed population prevalence of 50%, a 95% confidence level, and a 5% margin of error, resulting in a minimum required sample size of 402 participants [23]. A convenience sampling approach was employed. Personalized email invitations containing unique survey links were distributed to faculty members and students. Faculty were encouraged to share the survey with their students to increase participation. Up to three reminder emails were sent throughout the data collection period to improve response rates. To minimize duplicate responses, the survey platform restricted participation to one submission per unique email link.

### Data collection tool

Data were collected using a structured, self-administered online questionnaire via Qualtrics, developed by the authors [24]. The instrument included sections on sociodemographic characteristics, GenAI knowledge and awareness, patterns of adoption and use, and perceived benefits and barriers to GenAI integration in academic settings. Survey development was informed by a review of the literature and best practices in web-based survey design. The survey incorporated adaptive (skip-logic) functionality, such that participants were automatically directed to subsequent questions based on their prior responses, ensuring relevance and minimizing respondent burden. Response options included selections such as “Other” and “Prefer not to respond” to accommodate participants whose experiences were not fully captured by predefined categories or who chose not to disclose specific information. The full survey instrument is provided as Supplementary Material.

### Pilot testing

The questionnaire was piloted with three faculty members and four students before full deployment. Pilot feedback was used to evaluate the instrument’s clarity, completeness, relevance, acceptability, and cultural sensitivity. Revisions were made to improve item wording, response options, and overall survey flow before final administration.

### Data collection procedures

Data were collected electronically over three months (January–March 2025). Participation was voluntary, and no direct incentives were tied to survey completion. However, to enhance engagement, participants were offered entry into a random draw for one of fifty US$30 gift cards upon completion of the questionnaire.

### Statistical analysis

Survey data were exported and analyzed using IBM SPSS Statistics version 31 and Microsoft Excel [25, 26]. Univariate analyses were conducted to summarize participant characteristics and key study variables. Bivariate analyses were performed using Chi Square to assess associations between demographic variables and GenAI knowledge, adoption, and perceived benefits and barriers. Statistical significance was evaluated using a two-tailed alpha level of *p* < .05. Results are presented in tabular and narrative formats.

### Ethical considerations

The study adhered to established guidelines for the design and administration of web-based surveys, including confidentiality, voluntary participation, and data protection. Ethical approval was obtained from the California State University, Dominguez Hills Institutional Review Board (IRB-FY2025-66). All participants received an electronic information sheet describing the study objectives, procedures, potential risks and benefits, and their rights as participants. Informed consent was obtained from all of the participants and the study was performed in accordance with relevant guidelines and regulations (such as the Declaration of Helsinki).

## Results

Of the 559 participants who completed the questionnaire, 78.5% were students and 19.3% were faculty, 62.5% were female, and 57.5% were Hispanic/Latino. Participants were drawn from six academic colleges within the institution to reflect a broad cross-section of educational programs. However, 83.6% of respondents were affiliated with the College of Health, Human Services, and Nursing, which houses healthcare and allied health training programs, including disciplines directly involved in clinical practice, public health, and health services delivery (Table 1). The remaining participants were distributed across non-health colleges.

**Table 1:** Demographic profile of survey participants

Description	Number (%)
Role in School (*N* = 559)	
Faculty	108 (19.3)
Student	439 (78.5)
Administration or Management Staff	1 (0.2)
Other	11 (2.0%)
Sex at Birth (*N* = 558)	
Male	199 (35.7)
Female	349 (62.5)
I prefer not to say	10 (1.8)
Current affiliations (*N* = 557)	
Professional/Graduate Degree Programs	97 (17.4)
Undergraduate Degree Programs	438 (78.6)
I am not sure	22 (4.0)
Length of time in Current Unit (*N* = 557)	
Less than 5 years	451 (81.0)
5–9 years	68 (12.1)
10–14 years	18 (3.2)
15 and above	20 (3.6)
Race (*N* = 553)	
Hispanic/Latino	318 (57.5)
Not Hispanic/Latino	235 (42.5)
Ethnicity (*N* = 549)	
Black/African American	61 (11.1)
White/Caucasian	150 (27.3)
Native Hawaiian or Other Pacific Islander	2 (0.4)
Asian	86 (15.7)
Native American/Alaska Native	8 (1.5)
Mixed or Multiracial	56 (10.2)
Other	101 (18.4)
I prefer not to say	85 (15.5)
Highest Educational Qualification (*N* = 555)	
High School Diploma/GED	255 (46.0)
Associate degree	125 (22.5)
Bachelors	62 (11.2)
Masters	28 (5.1)
Doctorate	79 (14.2)
Other	6 (1.1)
College (*N* = 512)	
Arts and Humanities	11 (2.1)
Business Administration and Public Policy	14 (2.7)
Education	8 (1.6)
Continuing and Professional Education	1 (0.2)
Natural and Behavioral Sciences	15 (2.9)
Health, Human Services and Nursing	428 (83.6)
Others (Please Specify)	35 (6.8)

Approximately 33.2% of respondents reported being knowledgeable about their institution’s official use of GenAI applications, while 55.1% indicated that their institution or department uses GenAI as a learning support tool. Among the GenAI applications identified, learning assistants – most notably ChatGPT – were the most commonly used, reported by 84.2% of respondents (Table 2).

**Table 2:** GenAI knowledge among participants

Description	Number (%)
Knowledge of the institution’s official use of GenAI applications (*N* = 533)	
Definitely not	59 (11.1)
Probably not	118 (22.1)
Might or might not	197 (37.0)
Probably yes	126 (23.6)
Definitely yes	33 (6.2)
Knowledge of the department officially using any GenAI applications (*N* = 449)	
Definitely not	62 (13.8)
Probably not	138 (39.7)
Might or might not	175 (39.0)
Probably yes	62 (13.8)
Definitely yes	12 (2.7)
Knowledge of activities where GenAI is used in school (*N* = 356) *	
For learner support	196 (55.1)
Virtual patients/simulation practice	67 (18.8)
Personalized learning platforms (self-learning platform)	106 (29.8)
Tools for psychological support /individual issues for learners	46 (12.9)
Student management tools	114 (32.0)
Assessment and evaluation tools	96 (27.0
Research data analysis tools	133 (37.0)
Assignments, Quizzes, and Assessments	113 (31.7)
Report and Discussions	69 (19.4)
Editing and writing assignments	144 (40.5)
Scheduling and Calendaring	102 (28.7)
Knowledge of GenAI tools or applications (*N* = 506) *	
Learning Assistants (Chatbots: ChatGPT, Claude, CoPilot, Watson Assistant, etc.)	426 (84.2)
Learning Data Analysis (Coursera for Campus)	87 (17,2)
Learning Management Systems (Moodle with AI, Canvas with AI, etc.)	171 (33.8)
Fields where GenAI tools or applications are used currently (*N* = 506)	
I am not using any tools	170 (33.6)
Learning Assistants (Chatbots: ChatGPT, Claude, CoPilot, Watson Assistant, etc.)	290 (57.3)
Versions of GenAI tools participants use (*N* = 341)	
Entirely free tools	233 (68.3%)
Mostly paid tools	21 (6.2%)
Sources of funding for paid versions of GenAI tools (*N* = 101)	
Personal funds	67 (66.3)
University/College/Department	23 (22.8)
Understanding of GenAI tools or applications (*N* = 506)	
Learning Assistants	426 (84.2)
Learning Management Systems	171 (33.8)
Learning Data Analysis	87 (17.2)
I have not heard of any tools	60 (11.9)

*Multiple options allowed

Overall, 83.2% of respondents reported having used GenAI technologies, with 54.0% indicating use at least weekly. The most common applications included responding to assignments, quizzes, and assessments (40.4%) and designing or preparing lectures (24.1%). A large majority (81.2%) expressed satisfaction with GenAI use, and 42.3% perceived GenAI as very to extremely useful in supporting their academic activities. Despite high uptake and favorable perceptions, 19.7% of respondents reported experiencing difficulties with GenAI during at least half of their usage, while only 7.9% indicated having received any formal training on GenAI (Table 3).

**Table 3:** Participants’ use and exposure to GenAI

Description	Number (%)
Ever used GenAI technology (*N* = 547)	
No	50 (9.1)
Maybe	42 (7.7)
Yes	455 (83.2)
Frequency of use of GenAI tools and Applications (*N* = 331)	
Never	60 (18.1)
Several days per month	134 (40.5)
Several days per week	62 (18.7)
Weekly	40 (12.1)
Daily	35 (10.6)
Tasks where GenAI tools are used (*N* = 307)	
Responding to assignments, quizzes and assessments	124 (40.4)
Designing and developing training programs	49 (16.0)
Designing and preparing lectures	74 (24.1)
Designing and creating assessment activities (e.g., writing exam questions/cases, creating scenarios/scripts for exams)	82 (26.7)
Writing books and learning materials	46 (15.0)
Satisfaction with the GenAI tools or applications (*N* = 326)	
Extremely dissatisfied	7 (2.2)
Somewhat dissatisfied	21 (6.4)
Neither satisfied nor dissatisfied	66 (20.3)
Somewhat satisfied	179 (54.9)
Extremely satisfied	53 (16.3)
Skills improved by using GenAI in learning, teaching and research (*N* = 320) *	
Learning skills	196 (61.3)
Research skills	195 (60.9)
Collaboration skills	68 921.3)
Teaching skills	56 (17.5)
Curriculum design and development skills	59 (18.4)
Student assessment/evaluation skills	69 (21.6)
Technology utilization skills	83 (25.9)
Management skills	62 (19.4)
Impact of using GenAI on the quality of work (learning or teaching/research) (*N* = 324)	
Not at all useful	8 (2.5)
Slightly useful	56 (17.3)
Moderately useful	123 (38.0)
Very useful	99 (30.6)
Extremely useful	38 (11.7)
How often participants encounter difficulties while using GenAI in their learning or work (*N* = 326)	
Never	50 (15.3)
Sometimes	212 (65.0)
About half to most of the time	56 (17.2)
Always	8 (2.5)
Ever received training on the use of GenAI (*N* = 483)	
Yes, received training multiple times	13 (2.7)
Yes, received training once	25 (5.2)
No, never received training	429 (88.8)
Not sure	15 (3.1)
I prefer not to say	1 (0.2)
Researched regarding the application of GenAI (*N* = 484)	
None at all	174 (36.0)
A little	169 (34.9)
A moderate amount	101 (20.9)
A lot	24 (5.0)
Quite a lot	16 (3.3)

*Multiple options allowed

Common difficulties reported in the use of GenAI for learning or work included lack of formal training (45.3%), technological limitations (42.2%), and ethical or legal risks associated with GenAI use (34.6%). In addition, respondents expressed substantial concerns regarding the accuracy and reliability of GenAI-generated information (78.7%), insufficient training opportunities (71.3%), personal data privacy (64.9%), and the potential impact of GenAI on academic and research integrity (58.9%). Limited access to diverse GenAI tools and software was also reported by 57.9% of participants (Table 4). Despite these identified needs, 72.1% of respondents reported that they found GenAI tools useful across learning, teaching, and research activities.

**Table 4:** Attitude, sources, and benefits to GenAI tools and resources*

Description	Number (%)
Common difficulties encountered while using GenAI in your learning or work (*N* = 263)	
Time and workload pressure	51 (19.4)
Insufficient funds and equipment	34 (12.9)
Lack of training in GenAI	119 (45.3)
Technological limitations	111 (42.2)
Risks related to ethical/legal issues associated with GenAI	91 (34.6)
Common concerns participants have while using GenAI (*N* = 333)	
Personal data privacy	216 (64.9)
Accuracy and reliability of information	262 (78.7)
Loss of personalization in teaching	154 (46.3)
Decreased connection with students	114 (34.2)
Potential impact on academic and research integrity and honesty	196 (58.9)
Lack of regulations on the use of GenAI in the medical field	124 (37.2)
High costs for operation	65 (19.5)
Lack of skills to use GenAI effectively	115 (34.5)
Ethical or legal issues	187 (56.2)
Lack of training in GenAI	298 (71.3)
Poor access to various GenAI tools and software	242 (57.9)
Technical and technological support	216 (51.7)
Financial support	168 (40.2)
Encouragement from the school and hospitals	155 (37.1)
Reasons people use GenAI (*N* = 337)	
I find GenAI tools useful for learning, teaching and research	243 (72.1)
I enjoy exploring and using new technologies	154 (45.7)
I need to use it to adapt to GenAI changes that affect my work.	92 (27.3)
AI is a trendy topic, and I don’t want to lag behind	86 (25.5)
I see my colleagues using it and want to try it too	80 (23.7)
The school/work requires me to use it	57 (16.9)
Sources of GenAI information (*N* = 488)	
Traditional media (TV, newspapers, etc.)	127 (26.0)
Internet	377 (77.3)
Social media (e.g. Facebook, Instagram, TikTok, Zalo, Telegram, etc.)	272 (55.7)
Family and friends	167 (34.2)
Students/Colleagues	226 (46.3)
The University	111 (22.6)
Work partners	57 (11.7)
Benefits of applying GenAI to learning and research (*N* = 451)	
Improved teaching and research results	212 (47.0)
Enhanced learning experiences for learners	236 (52.3)
Timesaving	333 (72.8)
Expanded expertise knowledge	177 (39.3)
More opportunities to develop teaching, research, and professional skills	159 (35.3
Broadened knowledge and skills in technology	171 (37.9)
Better management of tasks and personal issues	141 (31.3)
Better support for students	131 (29.1)

*Multiple options allowed

Most respondents indicated that they primarily obtained GenAI-related information from online sources (77.3%), and 72.8% reported that GenAI use saved them time in their academic or professional activities (Table 4).

To support the effective integration and use of GenAI in learning, teaching, and research, most participants indicated a need for formal training in GenAI (71.3%), improved access to a broader range of GenAI tools and software (57.9%), and enhanced technical and technological support (51.7%).

Gender-based differences were observed across several GenAI-related perceptions and experiences. Male participants were significantly more likely to report knowledge of emerging technologies (*p* = .003), prior engagement in GenAI-related research (*p* = .013), and to perceive GenAI as an essential tool for learning, teaching, and research (*p* = .03). Males were also more likely to believe that GenAI has the potential to significantly influence users’ career trajectories (*p* < .001). In contrast, female participants were significantly more likely to indicate that GenAI training is necessary for healthcare education (*p* = .003) and to report greater overall concerns regarding GenAI use (*p* = .003). Additionally, a higher proportion of female respondents expressed concern that GenAI may replace healthcare professionals in the future, although this association did not reach conventional levels of statistical significance (*p* = .083) (Table 5).

**Table 5:** Perspective of generative artificial intelligence use and relevance

		Gender (Male/Females)										Ethnicity (Hispanic/Non-Hispanic						Role (Faculty/Student)					Academic Level (Graduate/Undergraduate)		
		Male: n (5)	Female: n (%)					Chi Square; (P-Value)				Hispanic: n (5)	Non-Hispanic: n (%)			Chi Square; (P-Value)		Faculty: n (%)	Students: n (%)			Chi Square; (P-Value)	Graduate: n(%)	Undergraduate: n (%)	Chi Square; (P-Value)
Heard of GenAI																									
Yes		192 (98)	342 (97.4)					7.9 (0.1)				299 (96.5)	233 (98.7)			3.2 (0.2)		103 (99)	432 (97)			2.2 (0.9)	92 (97.9)	407 (97.6)	4.1 (0.4)
Ever used GenAI Technologies																									
Yes		166 (84.3)	282 (82.7)					3.1 (0.53)				246 (79.4)	205 (88)			10.1 (0.01)		95 (91.4)	352 (81.7)			10.1 (0.12)	80 (86)	350 (83.7)	17.4 (0.002)
Understanding of GenAI Technologies																									
Moderate to Extensive		131 (74.4)	170 (55.1)								19.5 (0.003)	160 (59)	141 (67.1)			7.5 (0.06)		60 (61.9)		237 (62.7)		3.2 (0.96)	57 (67.1)	229 (62.1)	7.7 (0.26)
Ever received training on GenAI																									
Yes		18 (10.2)	20 (6.6)							19.5 (0.4)		14 (5.2)	22 (10.5)			9.2 (0.06)		18 (18.6)		18 (4.8)		28.9 (0.004)	12 (14.1)	25 (6.8)	12.6 (0.13)
Researched on GenAI																									
Moderate to Quite a lot		78 (44.3)	59 (19.4)							55.3 (<.001)		72 (26.6)	66 (31.4)			18.2 (0.001)		47 (48.5)		92 (24.3)		54.7 (<.001)	35 (41.2)	102 (27.6)	15.6 (0.05)
Knowledge of College/University using GenAI																									
Yes		64 (33.5)	92 (27.4)						8.3 (0.41)			66 (21.9)	91 (39.9)			23.7 (<.001)		50 (50)		104 (24.7)		30.1 (0.003)	43 (46.2)	109 (26.8)	24.5 (0.002)
Knowledge of Department using GenAI																									
Yes		26 (16.3)	48 (16.9)						5.6 (0.7)			33 (13.2)	40 (20.4)			5.6 (0.23)		17 (18.3)		56 (16.2)		19.4 (0.08)	17 (21.3)	55 (16.1)	5.3 (0.72)
Version of GenAI Used																									
Free version		75 (60.5)	158 (73.2)					11.1 (0.2)				133 (67.9)	98 (68.5)			4.9 (0.3)		51 (68)		177 (68.1)		15.1 (0.23)	57 (89.1)	235 (91.1)	19.0 (0.015)
Frequency of GenAI use for work																									
Daily to Weekly		62 (50.8)	75 (36.1)					10.1 (0.26)				67 (35.3)	69 (49.6)			15.3 (0.004)		39 (53.4)		96 (38.1)		28.6 (0.005)	30 (47.6)	103 (41.0)	3.9 (0.86)
Satisfaction with GenAI tools																									
Somewhat to extremely satisfied		91 (75.2)	140 (68.6)					7.7 (0.46)				125 (67.2)		107 (77.5)		6.4 (0.17)		52 (72.2)		176 (71)		11.3 (0.5)	46 (73)	246 (72)	18.3 (0.02)
Impact of using GenAI use on work quality																									
Very to Extremely Useful		102 (85)		157 (77.3)					9.5 (0.3)			145 (77.5)		114 (84.4)		4.2 (0.38)		57 (82.6)		198 (79.5)		5.9 (0.92)	28 (45.2)	106 (43.3)	14.3 (0.076)
Experience difficulties with GenAI																									
Most of the time to Always	104 (86)				171 (83.8)			7.1 (0.53)				153 (82.3)		121 (87.7)		2.6 (0.63)		60 (83.3)		212 (85.5)		6.7 (0.88)	4 (6.5)	17 (6.9)	6.4 (0.6)
Interested in learning more about GenAI																									
Probably to Definitely Yes	79 (45.4)			135 (43.1)				13.1 (0.11)				102 (35.9)		111 (53.9)		20.6 (<.001)		70 (73.7)		138 (35.8)		73.7 (<.001)	43 (50)	164 (43.6)	12.1 (0.15)
GenAI use is beneficial in learning, teaching and research																									
Yes		95 (55.6)					138 (45.3)				7.9 (0.09)	123 (44.7)			109 (53.4)	6.1 (0.05)		63 (66.3)		166 (44.4)		20.6 (0.002)	48 (55.8)	177 (48.4)	4.9 (0.3)
Have concerns about GenAI use in learning and research																									
Yes		58 (34.1)				117 (38.6)					7.9 (0.11)	83 (30.4)		94 (46.3)		12.6 (0.002)		49 (51.6)		126 (34)		12.4 (0.05)	40 (47.1)	130 (35.7)	10.7 (0.03)
GenAI use is necessary for training in healthcare																									
Probably to Definitely Yes		69 (42.6)				93 (32.6)					22.9 (0.003)	70 (27.6)		91 (46.7)		23.7 (<.001)		56 (61.5)		102 (29.3)		58.8 (<.001)	38 (44,7)	118 (34)	10.5 (0.24)
GenAI is an essential tool in learning, teaching and research																									
Somewhat to Strongly Agree		86 (53.1)				127 (44.9)					17.0 (0.03)	107 (42.3)		104 (53.6)		10.8 (0.029)		62 (68.9)		146 (42.1)		37.9 (<.001)	47 (55.3)	155 (44.9)	5.7 (0.68)
GenAI importance will increase in the future																									
Somewhat to Strongly Agree		115 (71)				201 (71)					18.4 (0.02)	175 (68.9)			141 (73.1)	5.4 (0.25)		73 (82)		237 (68.1)		26.7 (0.01)	60 (71.4)	246 (71.1)	11.0 (0.2)
Receiving training in GenAI is advantageous to one’s career																									
Somewhat to Strongly Agree		114 (70.4)				171 (60.2)					13.8 (0.09)	152 (59.8)			132 (68)	7.3 (0.12)		68 (75.6)		209 (60.1)		31.2 (0.002)	62 (72.9)	213 (61.6)	10.0 (0.27)
GenAI potential influence on career is very significant																									
Somewhat to Strongly Agree		115 (71)				161 (56.7)					47.3 (<.001)	141 (55.5)			133 (68.6)	12.1 (0.02)		70 (77.8)		198 (56.9)		31.0 (0.002)	61 (71.7)	206 (59.5)	9.8 (0.28)
Believe that all faculty should be trained on GenAI																									
Somewhat to Strongly Agree		99 (61.1)				159 (56.2)					9.5 (0.31)	137 (54.2)			119 (61.3)	11.8 (0.02)		63 (69.2)		186 (53.8)		28.4 (0.005)	54 (64.3)	197 (66.9)	10.9 (0.21)
Believe that all students should be trained on GenAI																									
Somewhat to Strongly Agree		115 (72.3)				171 (61.3)					17.2 (0.03)	153 (61)			132 (69.8)		11.5 (0.02)	72 (80.9)			208 (61)	41.9 (<.001)	60 (71.4)	215 (63.2)	7.7 (0.46)
Concerned that GenAI will replace faculty in the future																									
Probably to Definitely concerned		67 (42.1)				161 (57.7)					23.3 (0.003)	140 (56.2)			91 (47.6)		8.0 (0.09)	35 (38.9)			194 (57.1)	26.5 (0.01)	31 (36.9)	192 (56.3)	15.5 (0.05)
Concerned that GenAI will replace healthcare professionals in the future																									
Probably to Definitely concerned		64 (40.3)					147 (52.5)			20.6 (0.01)		130 (52.2			83 (43.2)		5.5 (0.24)	33 (36.7)			178 (52.2)	18.3 (0.11)	31 (36.9)	174 (51)	8.8 (0.36)

Ethnicity-based differences were observed across multiple GenAI-related domains. Non-Hispanic/Latino participants were significantly more likely to report prior use of emerging technologies (*p* = .006), awareness of their institution’s use of GenAI (*p* < .001), and interest in learning more about GenAI (*p* < .001). They were also more likely to agree that GenAI is beneficial for learning, teaching, and research (*p* = .047), to report concerns regarding GenAI use in academic contexts (*p* = .002), and to believe that GenAI is necessary for healthcare training (*p* < .001). In contrast, Hispanic/Latino participants were significantly more likely to report having researched GenAI at a moderate or high level (*p* = .001).

Role-based differences between faculty and students were also statistically significant. Faculty members were more likely to report having received formal training in GenAI (*p* = .004) and to have researched GenAI more extensively than students (*p* < .001). Faculty demonstrated greater knowledge of institutional GenAI use (*p* = .003) and reported more frequent use of GenAI for work-related activities (*p* = .005). Additionally, faculty expressed significantly higher interest in learning more about GenAI (*p* < .001) and were more likely to perceive GenAI as beneficial for learning, teaching, and research (*p* = .002).

Faculty also reported greater concerns regarding GenAI use in learning and research, were more likely to believe that GenAI is necessary for healthcare training (*p* < .001), and to view GenAI as an essential tool in learning, teaching, and research (*p* < .001). Furthermore, faculty were significantly more likely to recognize that the importance of GenAI will increase in the future (*p* = .009), to believe that GenAI training is advantageous for career development (*p* = .002), to perceive that GenAI has the potential to substantially influence career trajectories (*p* = .002), and to agree that all faculty members should receive GenAI training (*p* = .005).

Finally, academic level–based differences were observed in several GenAI-related competencies and perceptions. Professional and graduate scholars demonstrated significantly higher information technology proficiency (*p* = .007), were more likely to have used GenAI technologies (*p* = .002), and reported greater engagement in GenAI-related research (*p* = .049). This group also exhibited greater awareness of institutional GenAI use (*p* = .002), higher satisfaction with GenAI tools (*p* = .02), and greater concern regarding GenAI use in learning and research (*p* = .003). In contrast, undergraduate scholars were significantly more likely to report reliance on free versions of GenAI tools and resources (*p* = .015) and to express concern that GenAI may replace faculty in the future (*p* = .049) (Table 5). No statistically significant differences were observed in overall GenAI use by gender (*p* = .53) or by institutional role (faculty versus student; *p* = .12).

## Discussion

This study revealed high levels of use and perceived utility alongside substantial gaps in training, institutional awareness, and equitable access within a minority-serving institution in California. While patterns of use were broadly similar across gender and institutional role, differences emerged by ethnicity, academic level, and faculty status, suggesting uneven distribution of GenAI-related knowledge, exposure, and confidence. This finding aligns with previous research that also found differences in AI use between faculty and students, with these differences attributed to greater experience with technology among faculty [27]. AI use among students in our study exceeded that of a global study that reported that 86% of students regularly used AI for learning [28], but was lower than rates observed in a U.S. study, where 92% of students reported using AI in some form [29]. These findings position our sample between previously reported global and U.S. estimates, highlighting potential contextual differences in AI adoption.

In our study, 91.4% of faculty used AI technology and resources, with 53.4% using it at least weekly, a finding that is higher than those reported in prior studies [30, 31]. The most common uses for faculty in our study were to design course materials (22%), assist with administrative tasks like emails (16%), and create images (15%). The frequent use of AI among faculty contradicts previous research that demonstrated low use of AI among this group, and among faculty who used AI, did so to better understand the tools their students might be using [31]. Faculty AI use in our study extended beyond understanding student engagement with AI and instead demonstrated proactive integration into teaching and administrative practices. This suggests a shift from cautious observation to functional adoption.

A strong majority of participants in our study viewed GenAI as useful for learning, teaching, and research, with perceived benefits including time savings, enhanced learning, and improved research skills. These positive perceptions were particularly pronounced among faculty and male participants, who were also more likely to view GenAI as career-shaping. Positive perceptions of AI use among study participants was higher in our study compared to a previous study that documented 19% of instructors agreeing or strongly agreeing that GenAI was beneficial to teaching in their fields, with 56% of the respondents undecided [32]. Positive perceptions observed in our sample may reflect the growing integration of AI tools in academic workflows, where repeated use can reduce uncertainty and foster more pragmatic reflections of their usefulness. This finding suggests that perceptions of GenAI are dynamic and may continue to evolve as practical applications become more clearly established.

While our study found high uptake and favorable perceptions among study participants, we also found that formal preparation lagged substantially behind use. Fewer than one in ten respondents reported having received formal GenAI training, and fewer than one-third had engaged in sustained research on GenAI. Faculty and advanced scholars were more likely to have received training and to seek out GenAI knowledge independently, suggesting that experience and institutional longevity may facilitate exposure. This finding is in line with the current role gradient, where faculty/advanced scholars are more likely to seek training and independent knowledge, which fits diffusion-of-innovation dynamics, where more experienced institutional actors tend to have greater access to professional networks, committees, and early policy discussions, even when the institution has not yet scaled training to everyone [33]. Furthermore, the disconnect we found in individual experimentation, when compared to organizational readiness and communication, aligns with sector-wide evidence that institutions are still building the operating model (policy, training, support) while end users are already experimenting with significant gaps in AI policies/guidelines and organizational readiness [34, 35].

Satisfaction with GenAI tools was generally high, particularly among professional and graduate students, yet this satisfaction coexisted with widespread concerns and reported challenges. The combination of being highly satisfied while still reporting accuracy, privacy, integrity, and legal/ethical concerns from respondents aligns with the emerging literature that GenAI’s perceived productivity upside is real, but risk controls remain underdeveloped with policy ambiguity, assessment vulnerability, and the need for institution-level guardrails [36–38]. Most users in our study experienced difficulties related to a lack of training and technological limitations, and a large proportion expressed concerns about accuracy, data privacy, academic integrity, and ethical or legal implications. These concerns were more pronounced among faculty, non-Hispanic/Latino participants, and female respondents, indicating that higher engagement may coincide with greater critical awareness of GenAI risks.

Rapid developments in AI have intensified concerns about the potential displacement of human labor across multiple sectors [39]. We found that concerns about workforce displacement also existed among our study participants, but anxieties were specific to gender and roles within the university. Female respondents were more likely to express concern about GenAI replacing healthcare professionals, while undergraduate students were more likely to worry about GenAI replacing faculty. At the same time, a strong majority of participants believed GenAI would become essential to learning, teaching, and research over the next decade, and many expressed interest in additional training and institutional support. Our study underscores the need for professional development and clear guidelines to move AI adoption forward, as institutions need role-specific competencies, not generic awareness campaigns [40].

Taken together, the findings of this study point to an emerging GenAI divide within the institution by role, academic level, and ethnicity, characterized not by outright rejection of GenAI but by uneven access to training, tools, institutional guidance, and confidence. These findings align with other studies that reported group-specific differences in awareness, benefits, and utilization, reinforcing the current GenAI divide as an access-to-capability issue more than a pro and or anti-technology split [41, 42]. While both faculty and students in our study actively use GenAI, this use is occurring largely in the absence of structured support, coordinated policy communication, or embedded curricular integration. The convergence of high use, low training, strong perceived value, and persistent concern suggests that the institution is at an *inflection point*. Participants overwhelmingly articulated a need for formal training, broader access to GenAI tools, and technical support, indicating readiness for more deliberate, transparent, and equitable institutional engagement with GenAI. Without such action, existing disparities risk becoming institutionalized as GenAI adoption accelerates.

As GenAI continues to reshape higher education and healthcare systems, institutions must act deliberately to close emerging gaps in access, skills, and preparedness. By investing in inclusive training, infrastructure, and policy frameworks, higher education institutions can harness the transformative potential of GenAI while safeguarding equity, academic integrity, and long-term societal benefit. We recommend follow-on broader quantitative and qualitative studies to inform leadership decisions, the training of faculty and students on GenAI, the development of AI policies that are consistent with college and departmental values, as well as the creation of GenAI supporting environments with privacy-protective defaults, and clear “allowed vs not allowed” use cases in teaching, learning, and research to reduce ad hoc risk-taking and integrity confusion [36–36, 38, 42].

## Limitations

This study is subject to limitations inherent to cross-sectional, web-based survey designs. Selection bias is possible, as participation was restricted to individuals who voluntarily elected to respond. Additionally, because the total number of survey recipients could not be determined, a precise response rate could not be calculated. Data collection from a single institution further limits the generalizability of the findings to other higher education contexts.

The survey instrument did not include items assessing the acceptability of AI-focused training or coursework, which may have constrained the ability to fully evaluate readiness for formal GenAI integration into academic curricula. Inclusion of such measures in future studies would enable a more comprehensive assessment of attitudes toward AI education and provide actionable insights to inform curriculum design, particularly in health and professional training programs.

Although participants were recruited across six colleges to capture a broad range of academic disciplines, analyses were conducted at the institutional level. Departmental affiliation was deliberately excluded to preserve participant confidentiality, avoid potentially divisive comparisons, maintain institutional anonymity, and mitigate the risk of stigmatizing specific academic units. Moreover, the cross-sectional nature of the study precludes assessment of temporal trends or program-specific variation in GenAI adoption and perceptions.

Future research should adopt more systematic and purposive sampling strategies, coupled with deliberately proactive engagement approaches to enhance participation, representativeness, and internal validity. Building on the current findings, we plan to collaborate with multiple universities to conduct a multi-institutional study, leveraging this work as a baseline for longitudinal and comparative analyses that examine evolving GenAI adoption, training effectiveness, and equity implications across diverse academic settings.

## Conclusions

This study provides timely empirical evidence on the knowledge, use, perceptions, and challenges associated with generative artificial intelligence (GenAI) among faculty and students in higher education during a critical period of institutional policy development. Findings demonstrate widespread awareness and adoption of GenAI tools, accompanied by strong perceptions of their usefulness for learning, teaching, and research. At the same time, substantial gaps persist in formal training, institutional communication, and equitable access to GenAI resources.

Notably, disparities in GenAI engagement and preparedness were observed across academic role, level, and ethnicity, signalling an emerging AI divide that risks reinforcing existing structural inequities within higher education if left unaddressed. Faculty and advanced scholars exhibited higher levels of GenAI use, confidence, and institutional awareness, while undergraduate students and some racial and ethnic groups reported greater reliance on free tools, lower exposure to training, and heightened concerns about workforce displacement. Despite these challenges, there was a broad consensus that GenAI will become increasingly essential to academic and professional practice, particularly in healthcare and research-intensive fields.

The convergence of high utilization, limited formal training, and significant ethical, privacy, and integrity concerns underscores the need for coordinated institutional strategies to support responsible GenAI integration. These strategies should include transparent governance, structured training for both faculty and students, curriculum-embedded GenAI education, and policies that promote ethical, equitable, and effective use. Importantly, stakeholder engagement must be prioritized to address apprehensions, build trust, and ensure that GenAI adoption enhances, rather than undermines, educational quality and workforce readiness.

## Supplementary information

Supplementary Material 1.

Supplementary Material 2.

Supplementary Material 3.

Supplementary Material 4.

## Data Availability

The datasets used and/or analysed during the current study are available from the corresponding author on reasonable request.

## Abbreviations

AI: Artificial Intelligence
GenAI: Generative Artificial Intelligence
CA: California
CSU: California State University
CSUDH: California State University, Dominguez Hills
USA: United States of America
