# Supplementary material for: Generative artificial intelligence adoption and use in teaching and training healthcare professionals in higher education in the United States: a cross-sectional study
Source: BMC Med Educ. 2026 Apr 24;26:932. doi: 10.1186/s12909-026-09291-8 (PMC13238081; doi:10.1186/s12909-026-09291-8)
Supplement: Supplementary file 1 — Supplementary Material 1. [file 12909_2026_9291_MOESM1_ESM.docx]

**Supplement A:** Perspective of Generative Artificial Intelligence Use and Relevance by Biological Sex

| **Sex at Birth** | **Male N (%)** | **Females N (%)** | **Chi Square (df)** | ***P*-Value** |
| --- | --- | --- | --- | --- |
| ***Ability to Use Information Technology*** | |  |  |  |
| Good to Very Good | 168 (86.6) | 257 (78.4) | 9.9 (8) | .38 |
| Very Poor to Average | 26 (13.4) | 71 (21.6) |  |  |
| ***Heard of GenAI*** | | |  |  |
| Yes | 189 (97.9) | 321 (97.3) | 7.9 (4) | .1 |
| No/Maybe | 4 (2.1) | 9 (2.7) |  |  |
| ***Ever used GenAI Technologies*** |  |  |  |  |
| Yes | 163 (84.0) | 271 (82.4) | 3.1 (4) | .53 |
| No/Maybe | 31 (16.0) | 58 (17.6) |  |  |
| ***Understanding of GenAI Technologies*** | | | |  |
| Moderate to Extensive | 129 (74.1) | 163 (55.6) | 19.5 (6) | .003 |
| Limited to None | 45 (25.9) | 130 (44.4) |  |  |
| ***Ever received training on GenAI*** | | |  |  |
| Yes | 18 (10.4) | 19 (6.5) | 8.3 (8) | .4 |
| No/Not Sure | **155 (89.6)** | **273 (93.5)** |  |  |
| ***Researched on GenAI*** |  |  |  |  |
| Moderate to Quite a lot | 78 (44.8) | 57 (19.5) | 55.3 (8) | <.001 |
| A little to None | 96 (55.2) | 236 (80.5) |  |  |
| ***Knowledge of College/University using GenAI*** | |  |  |  |
| Yes | 64 (33.7) | 89 (27.4) | 8.3 (8) | .41 |
| No | 63 (33.2) | 105 (32.3) |  |  |
| Not sure | 63 (33.2) | 131 (40.3) |  |  |
| ***Knowledge of Department using GenAI*** | |  |  |  |
| Yes | 26 (16.5) | 47 (17.0) | 5.6 (8) | .7 |
| No | 66 (41.8) | 126 (45.5) |  |  |
| Not sure | 66 (41.8) | 104 (37.5) |  |  |
| ***Version of GenAI Used*** |  |  |  |  |
| Free version | 109 (89.3) | 191 (94.6) | 11.1 (8) | .2 |
| Paid version | 13 (10.7) | 11(5.4) |  |  |
| ***Frequency of GenAI use for work*** | | |  |  |
| Daily to Several days per week | 47 (38.5) | 49 (24,3) | 10.1 (8) | .26 |
| Weekly to several days per month | 56 (45.9) | 114 (56.4) |  |  |
| Never | 19 (15.6) | 39 (19.3) |  |  |
| ***Satisfaction with GenAI tools*** |  |  |  |  |
| Somewhat to extremely satisfied | 91 (75.2) | 134(67.7) | 7.7 (8) | .46 |
| Neither satisfied nor dissatisfied | 19 (15.7) | 47(23.7) |  |  |
| Somewhat to extremely dissatisfied | 11 (9.1) | 17(8.6) |  |  |
| ***Impact of using GenAI use on work quality*** | |  |  | .3 |
| Very to Extremely Useful | 62 (51.7) | 74 (37.6) | 9.5 (8) |  |
| Moderately Useful | 40 (33.3) | 79 (40.1) |  |  |
| Not Useful to Slightly Useful | 18 (15.0) | 44 (22.3) |  |  |
| ***Experience difficulties with GenAI*** | | |  |  |
| Most of the time to Always | 13 (10.7) | 12 (6.0) | 7.1 (8) | .53 |
| Sometimes to about half of the times | 91 (75.2) | 158 (78.6) |  |  |
| Never | 17 (14.0) | 31 (15,4) |  |  |
| ***Interested in learning more about GenAI*** | |  |  |  |
| Probably to Definitely Yes | 79 (45.9) | 132 (43.3) | 13.1 (8) | .11 |
| Might or Might Not | 45 (26.2) | 75 (24.6) |  |  |
| Probably to Definitely Not | 48 (27.9) | 98 (32.1) |  |  |
| ***GenAI use is beneficial in learning, teaching and research*** | | | |  |
| Yes | 94 (55.6) | 136 (45.6) | 7.9 (8) | .09 |
| Maybe | 58 (34.3) | 137 (46.0) |  |  |
| No | 17 (10.1) | 25 (8.4) |  |  |
| ***Have concerns about GenAI use in learning and research*** | | | |  |
| Yes | 57 (33.9) | 115 (38.9) | 7.6 (8) | .11 |
| Maybe | 52 (31.0) | 101 (34,1) |  |  |
| No | 59 (35.1) | 80 (27.0) |  |  |
| ***GenAI use is necessary for training in healthcare*** | | |  |  |
| Probably to Definitely Yes | 39 (29.5) | 93 (32.5) | 22.9 (8) | .003 |
| Might or Might Not | 51 (38.6) | 107 (37.4) |  |  |
| Probably to Definitely Not | 42 (31.8) | 86 (30.1) |  |  |
| ***GenAI is an essential tool in learning, teaching and research*** | | | |  |
| Somewhat to Strongly Agree | 86 (53.1) | 127 (44.9) | 17.0 (8) | .03 |
| Neither Agree nor Disagree | 51 (31.5) | 85 (30.0) |  |  |
| Somewhat to Strongly Disagree | 25 (15.4) | 71 (25.1) |  |  |
| ***GenAI importance will increase in the future*** | |  |  |  |
| Somewhat to Strongly Agree | 115 (71.0) | 201 (71.0) | 18.4 (8) | .02 |
| Neither Agree nor Disagree | 35 (21.6) | 47 (16.6) |  |  |
| Somewhat to Strongly Disagree | 12 (7.4) | 35 (12.4) |  |  |
| ***Receiving training in GenAI is advantageous to one’s career*** | | | |  |
| Somewhat to Strongly Agree | 114 (70.4) | 171 (60.2) | 13.8 (8) | .09 |
| Neither Agree nor Disagree | 29 (17.9) | 73 (25.7) |  |  |
| Somewhat to Strongly Disagree | 19 (11.7) | 40 (16,2) |  |  |
| ***GenAI potential influence on career is very significant*** | | |  |  |
| Somewhat to Strongly Agree | 115 (71.0) | 161 (56.7) | 47.3 (8) | <.001 |
| Neither Agree nor Disagree | 27 (16.7) | 77 (27.1) |  |  |
| Somewhat to Strongly Disagree | 20 (12.3) | 46 (16.2) |  |  |
| ***Believe that all faculty should be trained on GenAI*** | | |  |  |
| Somewhat to Strongly Agree | 99 (61.1) | 159 (56.2) | 9.5 (8) | .31 |
| Neither Agree nor Disagree | 42 (25.9) | 80 (27.1) |  |  |
| Somewhat to Strongly Disagree | 21 (13.0) | 44 (16.2) |  |  |
| ***Believe that all students should be trained on GenAI*** | | |  |  |
| Somewhat to Strongly Agree | 115 (72.3) | 171 (45.1) | 17.2 (8) | .028 |
| Neither Agree nor Disagree | 28(17.6) | 65(17.2) |  |  |
| Somewhat to Strongly Disagree | 16(10.1) | 143(37.7) |  |  |
| ***Concerned that GenAI will replace faculty in the future*** | | |  |  |
| Probably to Definitely concerned | 67(42.1) | 161(57.7) | 23.3 | .003 |
| Undecided | 33(20.8) | 62(22.2) |  |  |
| Probably to Definitely Not Concerned | 59(37.1) | 56(20.1) |  |  |
| Probably to Definitely concerned | 64(40.3) | 147(52.5) | 20.6 | .008 |
| Undecided | 21(13.2) | 56(20.0) |  |  |
| Probably to Definitely Not Concerned | 74(46.5) | 77(27.5) |  |  |

**Supplement B:** Perspective of Generative Artificial Intelligence Use and Relevance by Ethnicity

| **Ethnicity** | **Hispanic or Latino N (%)** | **Not Hispanic or Latino** **N (%)** | **Chi Square (df)** | ***P*-Value** |
| --- | --- | --- | --- | --- |
| ***Ability to Use Information Technology*** | |  |  |  |
| Good to Very Good | 336 (84.8) | 193 (82.8) | 2.7 (4) | .3 |
| Very Poor to Average | 60 (15.2) | 40 (17.2) |  |  |
| ***Heard of Generative Artificial Intelligence*** | |  |  |  |
| Yes | 285 (96.3) | 229 (99.1) | 3.2 (2) | .2 |
| No/Maybe | 11 (3.7) | 2 (0.9) |  |  |
| ***Ever used GenAI Technologies*** |  |  |  |  |
| Yes | 233 (78.7) | 204 (87.9) | 10.1 (2) | .006 |
| No/Maybe | 63 (21.3) | 28 (12.3) |  |  |
| ***Understanding of GenAI Technologies*** | |  |  |  |
| Moderate to Extensive | 151 (58.5) | 141 (67.1) | 7.5 (3) | .06 |
| Limited to None | 107 (41.5) | 69 (32.9) |  |  |
| ***Ever received training on GenAI*** | |  |  |  |
| Yes | 13 (5.1) | 22 (10.5) | 9.2 (4) | .06 |
| No/Not Sure | 244 (94.9) | 187 (89.5) |  |  |
| ***Researched on GenAI*** |  |  |  |  |
| Moderate to Quite a lot | 70 (37.2) | 66 (31.4) | 18.2 (4) | .001 |
| A little to None | 118 (62.8) | 144 (68.6) |  |  |
| ***Knowledge of College/University using GenAI*** | |  |  |  |
| Yes | 63 (21.8) | 91 (39.9) | 23.7 (4) | <.001 |
| No | 114 (39.4) | 55 (24.1) |  |  |
| Not sure | 112 (38.8) | 82 (36.0) |  |  |
| ***Knowledge of Department using GenAI*** | |  |  |  |
| Yes | 32 (13,2) | 40 (20.4) | 5.6 (4) | .23 |
| No | 114 (47.1) | 81 (41.3) |  |  |
| Not sure | 96 (39.7) | 75 (38.3) |  |  |
| ***Version of GenAI Used*** |  |  |  |  |
| Free version | 174 (94.6) | 126 (90.0) | 4.9 (4) | .3 |
| Paid version | 10 (5.4) | 14 (10.0) |  |  |
| ***Frequency of GenAI use for work*** | | |  |  |
| Daily to Several days per week | 44 (23.9) | 52 (37.4) | 15.3 (4) | .004 |
| Weekly to several days per month | 98 (53.3) | 71 (51.1) |  |  |
| Never | 42 (22.8) | 16 (11.5) |  |  |
| ***Satisfaction with GenAI tools*** |  |  |  |  |
| Somewhat to extremely satisfied | 119 (66.1) | 107 (77.5) | 6.4 (4) | .17 |
| Neither satisfied nor dissatisfied | 44 (24.4) | 21 (15.2) |  |  |
| Somewhat to extremely dissatisfied | 17 (9.4) | 10 (7.2) |  |  |
| ***Impact of using GenAI use on work quality*** | |  |  |  |
| Very to Extremely Useful | 73 (40.3) | 63 (46.7) | 4.2 (4) | .38 |
| Moderately Useful | 68 (37.6) | 51 (37.8) |  |  |
| Not Useful to Slightly Useful | 40 (22.1) | 21 (15.6) |  |  |
| ***Experience difficulties with GenAI*** | |  |  |  |
| Most of the time to Always | 12 (6.7) | 9 (6.5) | 2.6 (4) | .63 |
| Sometimes to about half of the times | 137 (76.1) | 112 (81.2) |  |  |
| Never | 31 (17.2) | 17 (12.3) |  |  |
| ***Interested in learning more about GenAI*** | |  |  |  |
| Probably to Definitely Yes | 99 (36.1) | 111 (53.9) | 20.6 (4) | <.001 |
| Might or Might Not | 80 (29.2) | 43 (20.9) |  |  |
| Probably to Definitely Not | 95 (34.7) | 52 (25.2) |  |  |
| ***GenAI use is beneficial in learning, teaching and research*** | | | |  |
| Yes | 120 (45.1) | 109 (53.4) | 6.1 (2) | .047 |
| Maybe | 125 (47.0) | 73 (35.8) |  |  |
| No | 21 (7.9) | 22 (10.8) |  |  |
| ***Have concerns about GenAI use in learning and research*** | | | |  |
| Yes | 80 (30.3) | 94 (46.3) | 12.6 (2) | .002 |
| Maybe | 96 (36.4) | 58 (28.6) |  |  |
| No | 88 (33.3) | 51 (25.1) |  |  |
| ***GenAI use is necessary for training in healthcare*** | | |  |  |
| Probably to Definitely Yes | 70 (27.6) | 91 (46.7) | 23.7 (4) | <.001 |
| Might or Might Not | 99 (39.0) | 58 (29.7) |  |  |
| Probably to Definitely Not | 85 (33.5) | 46 (23.6) |  |  |
| ***GenAI is an essential tool in learning, teaching and research*** | | | |  |
| Somewhat to Strongly Agree | 107 (42.3) | 104 (53.6) | 10.8 (4) | .029 |
| Neither Agree nor Disagree | 91 (36.0) | 47 (24.2) |  |  |
| Somewhat to Strongly Disagree | 55 (21.7) | 43 (22.2) |  |  |
| ***GenAI importance will increase in the future*** | |  |  |  |
| Somewhat to Strongly Agree | 175 (68.9) | 141 (73.1) | 5.4 (4) | .25 |
| Neither Agree nor Disagree | 54 (21.3) | 29 (15.0) |  |  |
| Somewhat to Strongly Disagree | 25 (9.8) | 23 (11.9) |  |  |
| ***Receiving training in GenAI is advantageous to one’s career*** | | | |  |
| Somewhat to Strongly Agree | 152 (59.8) | 132 (68.0) | 7.3 (4) | .123 |
| Neither Agree nor Disagree | 70 (27.6) | 33 (17.0) |  |  |
| Somewhat to Strongly Disagree | 32 (12.6) | 29 (14.9) |  |  |
| ***GenAI potential influence on career is very significant*** | | |  |  |
| Somewhat to Strongly Agree | 141 (55.5) | 133 (68.6) | 12.1 (4) | .017 |
| Neither Agree nor Disagree | 73 (28.7) | 32 (16.5) |  |  |
| Somewhat to Strongly Disagree | 40 (15.7) | 29 (14.9) |  |  |
| ***Believe that all faculty should be trained on GenAI*** | | |  |  |
| Somewhat to Strongly Agree | 137 (54.2) | 119 (61.3) | 11.8 (4) | .019 |
| Neither Agree nor Disagree | 78 (30.8) | 47 (24.2) |  |  |
| Somewhat to Strongly Disagree | 38 (15.0) | 28 (14.4) |  |  |
| ***Believe that all students should be trained on GenAI*** | | |  |  |
| Somewhat to Strongly Agree | 153 (61.4) | 132 (69.8) | 11.5 (4) | .022 |
| Neither Agree nor Disagree | 64 (25.7) | 30 (15.9) |  |  |
| Somewhat to Strongly Disagree | 32 (12.9) | 27 (14.3) |  |  |
| ***Concerned that GenAI will replace faculty in the future*** | | |  |  |
| Probably to Definitely concerned | 140 (56.2) | 91 (47.6) | 8.0 (4) | .093 |
| Undecided | 53 (21.3) | 42 (22.0) |  |  |
| Probably to Definitely Not Concerned | 56 (22.5) | 58 (30.4) |  |  |
| ***Concerned that GenAI will replace healthcare professionals in the future*** | | | |  |
| Probably to Definitely concerned | 13 0(52.2) | 83 (43.2) | 5.5 (4) | .237 |
| Undecided | 44 (17.7) | 33 (17.2) |  |  |
| Probably to Definitely Not Concerned | 75 (30.1) | 76 (39.6) |  |  |

**Supplement C:** Perspective of Generative Artificial Intelligence Use and Relevance by Role in College and University

| **Role in School** | **Faculty N (%)** | **Student N (%)** | **Chi Square (df)** | ***P*-Value** |
| --- | --- | --- | --- | --- |
| ***Ability to Use Information Technology*** | |  |  |  |
| Good to Very Good | 83 (83.0%) | 338 (80.5) | 10.2 (12) | .6 |
| Very Poor to Average | 17 (17.0) | 82 (19.5) |  |  |
| ***Heard of Generative Artificial Intelligence*** | | |  |  |
| Yes | 100 (99.0) | 407 (96.9) | 2.2 (6) | .9 |
| No/Maybe | 1 (1.0) | 13 (3.1) |  |  |
| ***Ever used GenAI Technologies*** |  |  |  |  |
| Yes | 92 (91.1) | 341 (81.4) | 10.1 (6) | .12 |
| No/Maybe | 9 (8.9) | 78 (18.6) |  |  |
| ***Understanding of GenAI Technologies*** | | |  |  |
| Moderate to Extensive | 58 (61.1) | 250 (64.6) | 3.2 (9) | .96 |
| Limited to None | 37 (38.9) | 137 (35.4) |  |  |
| ***Ever received training on GenAI*** | |  |  |  |
| Yes | 17 (17.9) | 18 (4.9) | 28.9 (12) | .004 |
| No/Not Sure | 78 (82.1) | 347 (95.1) |  |  |
| ***Researched on GenAI*** |  |  |  |  |
| Moderate to Quite a lot | 47 (48.5) | 92 (24.3) | 54.7 (12) | <.001 |
| A little to None | 50 (51.6) | 286 (75.7) |  |  |
| ***Knowledge of College/University using GenAI*** | |  |  |  |
| Yes | 49 (50.0) | 102 (24.9) | 30.1 (12) | .003 |
| No | 19 (19.4) | 146 (35.6) |  |  |
| Not sure | 30 (30.6) | 162 (39.5) |  |  |
| ***Knowledge of Department using GenAI*** | |  |  |  |
| Yes | 16 (17.6) | 56 (16.5) | 19.4 (12) | .08 |
| No | 50 (54.9) | 141 (41.6) |  |  |
| Not sure | 25 (27.5) | 142 (41.9) |  |  |
| ***Version of GenAI Used*** |  |  |  |  |
| Free version | 61 (83.6) | 134 (91.8) | 15.1 (12) | .23 |
| Paid version | 12 (16.4) | 12 (8.2) |  |  |
| ***Frequency of GenAI use for work*** | | |  |  |
| Daily to Several days per week | 27 (37.5) | 67 (27.1) | 28.6 (12) | .005 |
| Weekly to several days per month | 42 (58.3) | 126 (51.0) |  |  |
| Never | 3 (4.2) | 54 (21.9) |  |  |
| ***Satisfaction with GenAI tools*** |  |  |  |  |
| Somewhat to extremely satisfied | 51 (71.8) | 171 (70.4) | 11.3 (12) | .5 |
| Neither satisfied nor dissatisfied | 11 (15.5) | 54 (22.2) |  |  |
| Somewhat to extremely dissatisfied | 9 (12.7) | 18 (7.4) |  |  |
| ***Impact of using GenAI use on work quality*** | |  |  |  |
| Very to Extremely Useful | 32 (47.1) | 103 (42.2) | 5.9 (12) | .92 |
| Moderately Useful | 24 (35.3) | 92 (37.7) |  |  |
| Not Useful to Slightly Useful | 12 (17.6) | 49 (20.1) |  |  |
| ***Experience difficulties with GenAI*** | | |  |  |
| Most of the time always | 7 (9.9) | 15 (6.2) | 6.7 (12) | .88 |
| Sometimes to about half of the time | 52 (73.2) | 194 (80.5) |  |  |
| Never | 12 (16.9) | 32 (13.3) |  |  |
| ***Interested in learning more about GenAI*** | |  |  |  |
| Probably to Definitely Yes | 69 (75.0) | 136 (36.1) | 73.7 (12) | <.001 |
| Might or Might Not | 8 (8.7) | 110 (29.2) |  |  |
| Probably to Definitely Not | 15 (16.3) | 131 (34.7) |  |  |
| ***GenAI use is beneficial in learning, teaching and research*** | | | |  |
| Yes | 62 (66.7) | 164 (44.7) | 20.6 (6) | .002 |
| Maybe | 21 (22.6) | 172 (46.9) |  |  |
| No | 10 (10.8) | 31 (8.4) |  |  |
| ***Have concerns about GenAI use in learning and research*** | | | |  |
| Yes | 48 (51.6) | 124 (34.1) | 12.4 (6) | .05 |
| Maybe | 26 (28.0) | 123 (33.8) |  |  |
| No | 19 (20.4) | 117 (32.1) |  |  |
| ***GenAI use is necessary for training in healthcare*** | | |  |  |
| Probably to Definitely Yes | 56 (61.5) | 102 (29.3) | 58.8 (12) | <.001 |
| Might or Might Not | 23 (25.3) | 128 (36.8) |  |  |
| Probably to Definitely Not | 12 (13.2) | 118 (33.9) |  |  |
| ***GenAI is an essential tool in learning, teaching and research*** | | | |  |
| Somewhat to Strongly Agree | 62 (68.9) | 146 (42.1) | 37.9 (12) | <.001 |
| Neither Agree nor Disagree | 15 (16.7) | 118 (34.0) |  |  |
| Somewhat to Strongly Disagree | 13 (14.4) | 83 (23.9) |  |  |
| ***GenAI importance will increase in the future*** | |  |  |  |
| Somewhat to Strongly Agree | 73 (82.0) | 137 (55.2) | 26.7 (12) | .009 |
| Neither Agree nor Disagree | 10 (11.2) | 70 (28.2) |  |  |
| Somewhat to Strongly Disagree | 6 (6.7) | 41 (16.5) |  |  |
| ***Receiving training in GenAI is advantageous to one’s career*** | | | |  |
| Somewhat to Strongly Agree | 68 (75.6) | 209 (60.1) | 31.2 (12) | .002 |
| Neither Agree nor Disagree | 11 (12.2) | 89 (35.6) |  |  |
| Somewhat to Strongly Disagree | 11 (12.2) | 50 (14.4) |  |  |
| ***GenAI potential influence on career is very significant*** | | |  |  |
| Somewhat to Strongly Agree | 70 (77.8) | 198 (56.9) | 31.0 (12) | .002 |
| Neither Agree nor Disagree | 9 (10.0) | 92 (26.4) |  |  |
| Somewhat to Strongly Disagree | 11 (12.2) | 58 (16.7) |  |  |
| ***Believe that all faculty should be trained on GenAI*** | | |  |  |
| Somewhat to Strongly Agree | 63 (69.2) | 186 (53.8) | 28.4 (12) | .005 |
| Neither Agree nor Disagree | 15 (16.5) | 107 (30.9) |  |  |
| Somewhat to Strongly Disagree | 13 (14.3) | 53 (15.3) |  |  |
| ***Believe that all students should be trained on GenAI*** | | |  |  |
| Somewhat to Strongly Agree | 72 (80.9) | 208 (61.0) | 41.9 (12) | <.001 |
| Neither Agree nor Disagree | 10 (11.2) | 81 (23.8) |  |  |
| Somewhat to Strongly Disagree | 7 (7.9) | 52 (15.2) |  |  |
| ***Concerned that GenAI will replace faculty in the future*** | | |  |  |
| Probably to Definitely concerned | 35 (38.9) | 194 (57.1) | 26.5 (12) | .009 |
| Undecided | 19 (21.1) | 72 (21.2) |  |  |
| Probably to Definitely Not Concerned | 36 (40.0) | 74 (21.8) |  |  |
| ***Concerned that GenAI will replace healthcare professionals in the future*** | | | |  |
| Probably to Definitely concerned | 33 (36.7) | 178 (52.2) | 18.3 (12) | 0.11 |
| Undecided | 15 (16.7) | 58 (17.0) |  |  |
| Probably to Definitely Not Concerned | 42 (46.7) | 105 (30.8) |  |  |

**Supplement D:** Perspective of Generative Artificial Intelligence use and relevance by Level of Training (Graduate/Undergraduates)

| **Academic Block** | **Professional or Graduate N (%)** | **Undergraduate Degree N (%)** | **Chi-Square (df)** | ***P*-Value** | |
| --- | --- | --- | --- | --- | --- |
| ***Ability to Use Information Technology*** | |  |  |  | |
| Good to Very Good | 83 (83.3) | 334 (79.9) | 21.0 (8) | .007 | |
| Very Poor to Average | 11 (11.7) | 84 (20.1) |  |  | |
| ***Heard of Generative Artificial Intelligence*** | |  |  |  | |
| Yes | 92 (97.9) | 407 (97.6) | 4.1 (4) | .4 | |
| No/Maybe | 2 9 (2.1) | 10 (2.4) |  |  | |
| ***Ever used GenAI Technologies*** |  |  |  |  | |
| Yes | 80 (86.0) | 350 (83.7) | 17.4 (4) | .002 | |
| No/Maybe | 13 (14.0) | 68 (16.3) |  |  | |
| ***Understanding of GenAI Technologies*** | |  |  |  | |
| Moderate to Extensive | 57 (67.1) | 229 (62.1) | 7.7 (6) | .26 | |
| Limited to None | 28 (32.9) | 140 37.9) |  |  | |
| ***Ever received training on GenAI*** | |  |  |  | |
| Yes | 11 (13.1) | 25 (6.8) | 12.6 (8) | .13 | |
| No/Not Sure | 73 (86.9) | 342 (93.2) |  |  | |
| ***Researched on GenAI*** |  |  |  |  | |
| Moderate to quite a lot | 35 (41.2) | 102 (37.9) | 15.6 (8) | .049 | |
| A little to None | 50 (58.8) | 167 (62.1) |  |  | |
| ***Knowledge of College or University using GenAI*** | |  |  |  | |
| Yes | 43 (51.8) | 109 (26.8) | 24.5 (8) | .002 | |
| No | 13 (15.7) | 135 (33.3) |  |  | |
| Not sure | 27 (32.5) | 162 (39.9) |  |  | |
| ***Knowledge of Department using GenAI*** | |  |  |  | |
| Yes | 17 (21.3) | 55 (16.1) | 5.3 (8) | .72 | |
| No | 35 (43.8) | 150 (44.0) |  |  | |
| Not sure | 28 (35.0) | 136 (39.9) |  |  | |
| ***Version of GenAI Used*** |  |  |  |  | |
| Free version | 57 (89.1) | 235 (93.3) | 19.0 (8) | .015 | |
| Paid version | 7 (10.9) | 17 (6.7) |  |  | |
| ***Frequency of GenAI use for work*** | |  |  |  | |
| Daily to Several days per week | 22 (34.9) | 72 (28.7) | 3.9 (8) | .86 | |
| Weekly to several days per month | 33 (52.4) | 131 (52.2) |  |  | |
| Never | 8 (12.7) | 48 (19.2) |  |  | |
| ***Satisfaction with GenAI tools*** |  |  |  |  | |
| Somewhat to extremely satisfied | 46 (73.0) | 177 (72.0) | 18.3 (8) | .019 | |
| Neither satisfied nor dissatisfied | 14 (22.2) | 48 (19.5) |  |  | |
| Somewhat to extremely dissatisfied | 3 (4.8) | 21 (8.5) |  |  | |
| ***Impact of using GenAI use on work quality*** | |  |  |  | |
| Very to Extremely Useful | 28 (45.2) | 106 (43.3) | 14.3 (8) | .076 | |
| Moderately Useful | 22 (35,5) | 94 (38.4) |  |  | |
| Not Useful to Slightly Useful | 12 (19.4) | 45 (18.4) |  |  | |
| ***Experience difficulties with GenAI*** | |  |  |  | |
| Most of the time to Always | 4 (6.5) | 17 (6.9) | 6.4 (8) | .6 | |
| Sometimes to about half of the times | 47 (75.8) | 196 (79.4) |  |  | |
| Never | 11(17.7) | 34 (13.8) |  |  | |
| ***Interested in learning more about GenAI*** | |  |  |  | |
| Probably to Definitely Yes | 43 (50.0) | 164 (43.6) | 12.1 (8) | .15 | |
| Might or Might Not | 18 (20.9) | 98 (26.1) |  |  | |
| Probably to Definitely Not | 25 (29.1) | 114 (30.3) |  |  | |
| ***GenAI use is beneficial in learning, teaching and research*** | | | |  | |
| Yes | 48 (55.8) | 177 (48.4) | 4.9 (4) | .3 | |
| Maybe | 32 (37.2) | 154 (42.1) |  |  | |
| No | 6 (7.0) | 35 (9.6) |  |  | |
| ***Have concerns about GenAI use in learning and research*** | | | |  | |
| Yes | 40 (47.1) | 130 (35.7) | 10.7 (4) | .03 | |
| Maybe | 24 (28.2) | 127 (34.9) |  |  | |
| No | 21 (24.7) | 107 (29.4) |  |  | |
| ***GenAI use is necessary for training in healthcare*** | | |  |  | |
| Probably to Definitely Yes | 38 (44.7) | 118 (34.0) | 10.5 (8) | .24 | |
| Might or Might Not | 28 (32.9) | 126 (36.3) |  |  | |
| Probably to Definitely Not | 19 (22.4) | 103 (29.7) |  |  | |
| ***GenAI is an essential tool in learning, teaching and research*** | | | |  | |
| Somewhat to Strongly Agree | 47 (55.3) | 155 (44.9) | 5.7 (8) | .68 | |
| Neither Agree nor Disagree | 19 (22.4) | 114 (33.0) |  |  | |
| Somewhat to Strongly Disagree | 19 (22.4) | 76 (22.0) |  |  | |
| ***GenAI importance will increase in the future*** | |  |  |  | |
| Somewhat to Strongly Agree | 58 (79.7) | 246 (71.1) | 11.0 (8) | .2 | |
| Neither Agree nor Disagree | 14 (17.1) | 66 (19.1) |  |  | |
| Somewhat to Strongly Disagree | 10 (12.2) | 34 (9.8) |  |  | |
| ***Receiving training in GenAI is advantageous to one’s career*** | | | |  | |
| Somewhat to Strongly Agree | 62 (72.9) | 213 (65.3) | 10.0 (8) | .27 | |
| Neither Agree nor Disagree | 13 (15.3) | 84 (25.8) |  |  | |
| Somewhat to Strongly Disagree | 10 (11.8) | 29 (8.9) |  |  | |
| ***GenAI potential influence on career is very significant*** | | |  |  | |
| Somewhat to Strongly Agree | 61(71.8 | 206 (59.5) | 9.8 (8) | .28 | |
| Neither Agree nor Disagree | 17 (20.0) | 81 (23.4) |  |  | |
| Somewhat to Strongly Disagree | 7 (8.2) | 59 (17.1) |  |  | |
| ***Believe that all faculty should be trained on GenAI*** | | |  |  | |
| Somewhat to Strongly Agree | 54 (64.3) | 197 (56.9) | 10.9 (8) | .21 | |
| Neither Agree nor Disagree | 19 (22.6) | 96 (27.7) |  |  | |
| Somewhat to Strongly Disagree | 11 (13.1) | 53 (15.3) |  |  | |
| ***Believe that all students should be trained on GenAI*** | | |  |  | |
| Somewhat to Strongly Agree | 60 (71.4) | 215 (63.2) | 7.7 (8) | .46 | |
| Neither Agree nor Disagree | 14 (16.7) | 77 (22.6) |  |  | |
| Somewhat to Strongly Disagree | 10 (11.9) | 48 (14.1) |  |  | |
| ***Concerned that GenAI will replace faculty in the future*** | | |  |  | |
| Probably to Definitely concerned | 31 (36.9) | 192 (56.3) | 15.5 (8) | .049 | |
| Undecided | 22 (26.2) | 67 (19.6) |  |  | |
| Probably to Definitely Not Concerned | 31 (36.9) | 82 (24.0) |  |  | |
| ***Concerned that GenAI will replace healthcare professionals in the future*** | | | |  |  |
| Probably to Definitely concerned | 31 (36.9) | 174 (51.0) | 8.8 (8) | .36 | |
| Undecided | 19 (22.6) | 54 (15.8) |  |  | |
| Probably to Definitely Not Concerned | 34 (40.5) | 113 (33.1) |  |  | |
