# Supplementary material for: Generative artificial intelligence adoption and use in teaching and training healthcare professionals in higher education in the United States: a cross-sectional study
Source: BMC Med Educ. 2026 Apr 24;26:932. doi: 10.1186/s12909-026-09291-8 (PMC13238081; doi:10.1186/s12909-026-09291-8)
Supplement: Supplementary file 2 — Supplementary Material 2. [file 12909_2026_9291_MOESM2_ESM.docx]

Faculty/Students’ Perspectives, Knowledge, Attitudes, and Practices of Artificial Intelligence (AI

Start of Block: Information Sheet

**California State University, Dominguez Hills
 Study Information Sheet**
 **Introduction**:
 You are being asked to participate in a research study conducted by Dr Obinna Oleribe (DrPH, MBA, MBBS), Dr Matt Mutchler (Ph.D.), and Dr Parichart Sabado, (PhD, MPH), from the School of Public Health and Health Sciences, College of Health, Human Services and Nursing at California State University, Dominguez Hills. As a faculty member/student over 18 years of age in the College of Health, Human Services, and Nursing, you are invited to participate in this study as your participation will help generate a College-wide view of the concepts.

 **Purpose and Description of the Study:**
 The purpose of this study is to document the perspectives, knowledge, attitudes, and practices of faculty/students in the College of Health, Human Services, and Nursing to artificial intelligence (AI). If you decide to participate in this study, you will complete a self-administered questionnaire (survey). The survey has questions that explore your knowledge, understanding, and use of AI. We will also collect some demographic information, but no personally identifiable data will be collected. The survey should take approximately 15 minutes to complete.

 **Risk(s) and Discomfort(s):**
 There are no risk(s) associated with this study. Participation in this study is voluntary and if you volunteer to participate in this study, you may withdraw at any time without any consequences. You may also refuse to answer any questions you don’t want to answer and still remain in the study.

 **Benefits:**
 By participating in this study, you will contribute to science, advance AI evolution, and provide vital information for evidence-based decisions.

 **Confidentiality:**
 The study is confidential, so any information provided cannot be traced back to you. Please do not include your name or other identifying information in your survey responses that can identify you. Data will be analyzed in aggregate and stored in a secure location outside the reach of non-investigators.

 **Contact information:**
 If you have any questions or concerns about the research, please feel free to contact Obinna Oleribe at *ooleribe@csudh.edu*. If you have questions regarding your rights as a research participant, contact the California State University, Dominguez Hills IRB Office at 310-243-3756 or *irb@csudh.edu.*

 **This study information sheet is for you to keep.** Please save or print a copy of this page for your records or take a screenshot of it. Do you agree to participate

- Yes (1)
- No (2)

Skip To: End of Survey If California State University, Dominguez Hills Study Information Sheet Introduction: You are being... = No

End of Block: Information Sheet

Start of Block: A. Background and Demography

Q1 What is your role in the school?

- Faculty (1)
- Students (2)
- Admin or Management Staff (3)
- Others (please specify) (4) __________________________________________________

Q2 What is your gender at birth

- Male (1)
- Female (2)
- Non-binary / third gender (3)
- Prefer not to say (4)

Q3 What is your current college of study?

- Arts and Humanities (1)
- Business Administration and Public Policy (2)
- Education (3)
- Continuing and Professional Education (4)
- Natural and Behavioral Sciences (5)
- Health, Human Services and Nursing (6)
- Others (Please Specify) (7) __________________________________________________

Q4 Which block does the department you are working or schooling in belong to?

- Professional Degree Programs (1)
- Undergraduate Degree Programs (2)
- I am not sure (3)

Q5 How long have you been working or schooling at a College of Health, Human Services and Nursing

- Less than 5 years (1)
- 5 - 9 years (2)
- 10 - 14 years (3)
- 15 - 19 years (4)
- 20 years or more (5)

Q6 What is your ethnicity?

- Hispanic/Latino (1)
- Not Hispanic/Latino (2)

Q7 What is your race?

- Black/African American (1)
- White/Caucasian (2)
- Native Hawaiian or Other Pacific Islander (3)
- Asian (4)
- Native American/Alaska Native (5)
- Mixed or Multiracial (6)
- Others (7) __________________________________________________
- I prefer not to say (8)

Q8 What is your highest educational qualification?

- High School Diploma/GED (1)
- Associate Degree (2)
- Bachelors (3)
- Masters (4)
- Doctorate (5)
- Others (6) __________________________________________________

End of Block: A. Background and Demography

Start of Block: B. Knowledge and Perspectives

Q9 How would you assess your ability to us information technology?

- Terrible (1)
- Poor (2)
- Average (3)
- Good (4)
- Excellent (5)

Q10 Have you heard of Artificial Intelligence (AI)?

- No (1)
- Maybe (2)
- Yes (3)

Q11 Have you ever used AI technology?

- No (1)
- Maybe (2)
- Yes (3)

Q12 How would you assess your understanding of AI?

- Nver heard of AI (1)
- Heard of the term but not sure what it is (2)
- Limited understanding of AI (Have tried using a few AI applications) (3)
- Moderate understanding of AI (Frequently use many AI applications and have knowledge about the related technologies) (4)
- Extensive knowledge of AI (Understand AI technology and have participated in developing AI applications) (5)

Q13 Have you ever received training on the use of AI in medical education?

- Yes, received training multiple times (1)
- Yes, received training once (2)
- No, never received training (3)
- Maybe (4)
- I prefer not to say (5)

Q14 How much have you researched regarding the application of AI in your field of expertise?

- None at all (1)
- A little (2)
- A moderate amount (3)
- A lot (4)
- A great deal (5)

Q15 Do you know if CSUDH is officially using any AI applications?

- Definitely not (1)
- Probably not (2)
- Might or might not (3)
- Probably yes (4)
- Definitely yes (5)

Skip To: Q18 If Do you know if CSUDH is officially using any AI applications? = Definitely not

Q16 If using, do you know if your department is officially using any AI applications?

- Definitely not (1)
- Probably not (2)
- Might or might not (3)
- Probably yes (4)
- Definitely yes (5)

Q17 If using, in which activities is AI used at CSUDH? (Select all that apply)

- For learner support (1)
- Virtual patients/simulation practice (2)
- Personalized learning platforms (self-learning platform) (3)
- Tools for psychological support /individual issues for learners (4)
- Student management tools (5)
- Assessment and evaluation tools (6)
- Research data analysis tools (7)
- Assignments, Quizzes and Assessments (8)
- Report and Discussions (9)
- Editing and writing assignments (10)
- Scheduling and Calendaring (11)
- Others (please specify) (12) __________________________________________________

Q18 Have you heard of any of the following AI tools or applications? (Select all that apply)

- I have not heard of any tools (1)
- Learning Assistants (Chatbots: ChatGPT, Claude, CoPilot, Watson Assistant, etc.) (2)
- Medical Simulation Software (SimX, OssoVR, etc.) (3)
- Learning Data Analysis (Coursera for Campus) (4)
- Learning Management Systems (Moodle with AI, Canvas with AI, etc.) (5)
- Diagnostic and Treatment Assistant (VisualDx, IBM Watson, Touch Surgery, etc.) (6)
- Other (please specify below) (7) __________________________________________________

Q19 In which of the following fields are you currently using AI tools or applications? (Select all that apply)

- I am not using any tools (1)
- Learning Assistants (Chatbots: ChatGPT, Claude, CoPilot, Watson Assistant, etc.) (2)
- Medical Simulation Software (SimX, OssoVR, etc.) (3)
- Learning Data Analysis (Coursera for Campus) (4)
- Learning Management Systems (Moodle with AI, Canvas with AI, etc.) (5)
- Assessment and feedback systems for learners (e.g., Gradescope) (6)
- Diagnostic and Treatment Assistant (VisualDx, IBM Watson, Touch Surgery, etc.) (7)
- Other (please specify below) (8) __________________________________________________

Q20 What are your reasons for using AI? (Select all that apply

- I enjoy exploring and using new technologies (1)
- AI is a trendy topic, and I don't want to lag behind (2)
- I see my colleagues using it and want to try it too (3)
- The school/work requires me to use it (4)
- I find AI tools useful for learning, teaching and research (5)
- I need to use it to adapt to AI changes that affect my work. (6)
- Other (please specify below) (7) __________________________________________________

Q21 What versions of the AI tools do you use?

- Entirely free tools (1)
- Mostly free tools (2)
- Mostly paid tools (3)
- Entirely paid tools (4)
- Not using any AI tool (5)

Skip To: Q23 If What versions of the AI tools do you use? = Entirely free tools

Q22 If you use paid versions of AI tools, where does the funding come from? (Select all that apply)

- Personal funds (1)
- University/College (2)
- Department/Unit (3)
- Research funding units (4)
- Other (please specify below) (5) __________________________________________________

Q23 How often do you use AI tools in your work?

- Never (1)
- Several days per month (2)
- Several days per week (3)
- Weekly (4)
- Daily (5)

Q24 In which tasks do you use AI tools? (Select all that apply)

- Responding to assignments, quizzes and assessments (1)
- Designing and developing training programs (2)
- Designing and preparing lectures (3)
- Designing and creating assessment activities (e.g., writing exam questions/cases, creating scenarios/scripts for exams) (4)
- Writing books and learning materials (5)
- Using AI-integrated tools to train students in medical skills in simulation environments (e.g., interacting with virtual patients, simulated surgeries, practicing with virtual reality/augmented reality tools) (6)
- Monitoring student progress and providing feedback/assessments (7)
- Managing student data (8)
- Career counseling for students (9)
- Literature review for research (10)
- Data analysis in research (11)
- Conducting research experiments (12)
- Using telemedicine, remote learning (virtual classrooms, online exams, etc.) (13)
- Managing or planning teaching activities of the department (14)
- Synthesizing information to make clinical decisions (e.g., supporting diagnosis, treatment planning, analyzing test results) (15)
- Other (please specify below) (16) __________________________________________________

Q25 How would you rate your satisfaction with the AI tools or applications you have used?

- Extremely dissatisfied (1)
- Somewhat dissatisfied (2)
- Neither satisfied nor dissatisfied (3)
- Somewhat satisfied (4)
- Extremely satisfied (5)

Q26 Which of your skills do you feel have been improved by using AI in learning, teaching and research? (Select all that apply)

- Learning skills (1)
- Research skills (2)
- Collaboration skills (3)
- Clinical expertise (4)
- Teaching skills (5)
- Curriculum design and development skills (6)
- Student assessment/evaluation skills (7)
- Technology utilization skills (8)
- Management skills (9)
- Other (please specify below) (10) __________________________________________________

Q27 How would you assess the impact of using AI on the quality of your work (learning or teaching/research)?

- Not at all useful (1)
- Slightly useful (2)
- Moderately useful (3)
- Very useful (4)
- Extremely useful (5)

Q28 Do you encounter difficulties when using AI in your learning or work?

- No (1)
- Maybe (2)
- Yes (3)

Skip To: Q30 If Do you encounter difficulties when using AI in your learning or work? = No

Q29 If yes, what difficulties do you encounter when using AI in your work? (Select all that apply)

- Time and workload pressure (1)
- Insufficient funds and equipment (2)
- Lack of training in AI (3)
- Technological limitations (4)
- Risks related to ethical/legal issues associated with AI (5)
- Other (please specify below) (6) __________________________________________________

Q30 Where do you get information about AI? (Select all that apply)

- Traditional media (TV, newspapers, etc.) (1)
- Internet (2)
- Social media (Facebook, Instagram, TikTok, Zalo, Telgram) (3)
- Family and friends (4)
- Students/Colleagues (5)
- The University (6)
- Work partners (7)
- Other (please specify below) (8) __________________________________________________

Q31 Are you currently interested in learning more about and receiving training in using AI for learning, teaching and research?

- Definitely not (1)
- Probably not (2)
- Might or might not (3)
- Probably yes (4)
- Definitely yes (5)

Skip To: Q33 If Are you currently interested in learning more about and receiving training in using AI for learni... = Definitely yes

Q32 If not interested, what are the reasons you are not interested or still undecided about receiving information and training in applying AI to your teaching and research?(Select all that apply)

- I think it's not important (1)
- 0I do not have time (2)
- I am hesitant to try new things (3)
- I don’t have enough technological skills (4)
- 0I don't have sufficient resources and equipment (computers, phones, etc.) (5)
- I have concerns about the safety and accuracy of AI tools (6)
- I am worried about ethical/legal issues related to AI (7)
- Other (please specify below) (8) __________________________________________________

Q33 Do you think using AI in learning, teaching and research is beneficial to you?

- No (1)
- Maybe (2)
- Yes (3)

Q34 What benefits does applying AI to learning and research provide for you? (Select all that apply)

- Improved teaching and research results (1)
- Enhanced learning experiences for learners (2)
- Time-saving (3)
- Expanded expertise knowledge (4)
- More opportunities to develop teaching, research, and professional skills (5)
- Broadened knowledge and skills in technology (6)
- Better management of tasks and personal issues (7)
- Better management and support for students (8)
- Other (please specify below) (9) __________________________________________________

Q35 Do you have any concerns about using AI in your learning and research?

- No (1)
- Maybe (2)
- Yes (3)

Skip To: End of Block If Do you have any concerns about using AI in your learning and research? = No

Q36 If yes, hat concerns do you have about using AI in your learning, teaching, and research? (Select all that apply)

- Personal data privacy (1)
- Accuracy and reliability of information (2)
- Loss of personalization in teaching (3)
- Decreased connection with students (4)
- Potential impact on academic and research integrity and honesty (5)
- Lack of regulations on the use of AI in the medical field (6)
- High costs for operation (7)
- Lack of skills to use AI effectively (8)
- Ethical or legal issues (9)
- High costs for operation (10)
- Lack of skills to use AI effectively (11)
- Slow adaptation to the ongoing changes in science and technology (12)
- Other (please specify below) (13) __________________________________________________

End of Block: B. Knowledge and Perspectives

Start of Block: C. The Necessity and Recommendations

Q37 Do you think using AI is necessary for training in health, human services, and nursing schools?

- Definitely not (1)
- Probably not (2)
- Might or might not (3)
- Probably yes (4)
- Definitely yes (5)

Q38 Do you agree that AI is now an essential tool in learning, teaching, and research activities in health, human services, and nursing school?

- Strongly disagree (1)
- Somewhat disagree (2)
- Neither agree nor disagree (3)
- Somewhat agree (4)
- Strongly agree (5)

Q39 Do you agree that in the next 5-10 years, AI will become an essential tool in learning, teaching and research activities in health, human services, and nursing school?

- Strongly disagree (1)
- Somewhat disagree (2)
- Neither agree nor disagree (3)
- Somewhat agree (4)
- Strongly agree (5)

Q40 Do you agree that receiving training in AI will benefit your future career?

- Strongly disagree (1)
- Somewhat disagree (2)
- Neither agree nor disagree (3)
- Somewhat agree (4)
- Strongly agree (5)

Q41 Do you agree that the potential for AI applications in the field you are learning, teaching and researching is very significant?

- Strongly disagree (1)
- Somewhat disagree (2)
- Neither agree nor disagree (3)
- Somewhat agree (4)
- Strongly agree (5)

Q42 According to you, in which areas is AI likely to be widely applied? (Select all that apply)

- Studying/Learning (1)
- Assignments (2)
- Patents' Diagnosis (3)
- Decision-making for treatment (4)
- Consultation and treatment supervision (5)
- Theoretical teaching (6)
- Practical teaching (7)
- Student assessment (8)
- Management and organization of training (9)
- Research (10)
- Other (please specify below) (11) __________________________________________________

Q43 Do you believe that all faculty should receive training about AI and its applications in health and nursing education?

- Strongly disagree (1)
- Somewhat disagree (2)
- Neither agree nor disagree (3)
- Somewhat agree (4)
- Strongly agree (5)

Q44 What content do you think should be included in a course on AI and its applications in medicine for faculty?(Select all that apply)

- Basic knowledge on AI (1)
- Types of AI applications in health and nursing education (2)
- Types of AI applications in clinical practice (3)
- Ethical and legal issues related to AI (4)
- Development and evaluation of algorithms (5)
- Programming skills (Coding) (6)
- Writing prompts (7)
- Evaluate the quality of the product obtained from using the AI application (8)
- Other (please specify below) (9) __________________________________________________

Q45 Do you believe that all students should be taught about AI and its applications in their field of study?

- Strongly disagree (1)
- Somewhat disagree (2)
- Neither agree nor disagree (3)
- Somewhat agree (4)
- Strongly agree (5)

Q46 What content do you think should be included in a course on AI and its applications for students?(Select all that apply)

- Basic knowledge on AI (1)
- Types of AI applications in health and nursing education (2)
- Types of AI applications in clinical practice (3)
- Ethical and legal issues related to AI (4)
- Development and evaluation of algorithms (5)
- Programming skills (Coding) (6)
- Writing prompts (7)
- Evaluate the quality of the product obtained from using the AI application (8)
- Other (please specify below) (9) __________________________________________________

Q47 Are you concerned that in the future, AI might replace faculty members?

- Definitely not concerned (1)
- Probably not concerned (2)
- Undecided (3)
- Probably concerned (4)
- Definitely concerned (5)

Q48 Are you concerned that in the future, AI might replace healthcare professionals?

- Definitely not concerned (1)
- Probably not concerned (2)
- Undecided (3)
- Probably concerned (4)
- Definitely concerned (5)

Q49 What support do you need to effectively integrate the use of AI tools into your learning, teaching, and research activities in health, human services and nursing school? (Select all that apply)

- Training in AI (1)
- Easy access to various AI tools and software (2)
- Technical and technological support (3)
- Financial support (4)
- Encouragement from the school and hospitals (5)
- Other (please specify below) (6) __________________________________________________

Q50 Do you have any suggestions for enhancing the application of AI in health, human services, and nursing education at CSUDH?

________________________________________________________________

Q51 What did we miss? Please add here

________________________________________________________________

End of Block: C. The Necessity and Recommendations
