# Supplementary material for: Generative artificial intelligence adoption and use in teaching and training healthcare professionals in higher education in the United States: a cross-sectional study
Source: BMC Med Educ. 2026 Apr 24;26:932. doi: 10.1186/s12909-026-09291-8 (PMC13238081; doi:10.1186/s12909-026-09291-8)
Supplement: Supplementary file 3 — Supplementary Material 3. [file 12909_2026_9291_MOESM3_ESM.pdf]

Outlook

IRB-FY2025-66 - Initial: Exempt Determination

From do-not-reply@cayuse.com <do-not-reply@cayuse.com>
Date Wed 11/6/2024 8:40 AM
To Obinna Oleribe <ooleribe@csudh.edu>

Date:	November 6, 2024
Decision:	Exempt
Protocol Number:	IRB-FY2025-66
Title of the Study	Faculty/Students' Perspectives, Knowledge, Attitudes, and Practices of Artificial Intelligence (AI) in Health Sciences and Nursing Education
Principal Investigator	Obinna Oleribe

Dear Obinna Oleribe,

The California State University, Dominguez Hills Institutional Review Board is pleased to inform you that **your proposed study was determined to be exempt**, according to the federal regulations 45 CFR 46 since it meets the following criteria: Category 2.(i). Research that only includes interactions involving educational tests (cognitive, diagnostic, aptitude, achievement), survey procedures, interview procedures, or observation of public behavior (including visual or auditory recording) if at least one of the following criteria is met:
The information obtained is recorded by the investigator in such a manner that the identity of the human subjects cannot readily be ascertained, directly or through identifiers linked to the subjects; Category 2.(ii). Research that only includes interactions involving educational tests (cognitive, diagnostic, aptitude, achievement), survey procedures, interview procedures, or observation of public behavior (including visual or auditory recording) if at least one of the following criteria is met:
Any disclosure of the human subjects' responses outside the research would not reasonably place the subjects at risk of criminal or civil liability or be damaging to the subjects' financial standing, employability, educational advancement, or reputation; or
.

Your protocol has been assigned the following identification number: IRB-FY2025-66. Please retain your IRB protocol number and refer to it in any correspondences with the IRB regarding this study.

The protection of human subjects is our top priority. To ensure that all of your research activities are in compliance with federal regulations protecting human subjects:

1. Make sure that all subject-facing documents that you intend to distribute (e.g., flyers, announcements, recruitment emails, consent forms, and surveys) are the most up-to-date

versions that have been approved by the IRB. These are available in Cayuse under "Protocol Attachments."
2. Report all procedural changes and submit all protocol amendments to the IRB. No changes to protocol can be made without IRB approval, unless actions were taken to eliminate immediate hazards. Notify the IRB if there are any adverse effects that were a result of your study.
3. Notify the IRB when **all** research activities pertaining to the study (recruitment, data collection, and data analysis) are complete.

If you have any questions, please contact the IRB at irb@csudh.edu.

Sincerely,

Judith Aguirre, CIP
Research Compliance Officer
CSUDH Institutional Review Board

CSUDH | GRADUATE STUDIES & RESEARCH
