# Supplementary material for: Generative artificial intelligence adoption and use in teaching and training healthcare professionals in higher education in the United States: a cross-sectional study
Source: BMC Med Educ. 2026 Apr 24;26:932. doi: 10.1186/s12909-026-09291-8 (PMC13238081; doi:10.1186/s12909-026-09291-8)
Supplement: Supplementary file 4 — Supplementary Material 4. [file 12909_2026_9291_MOESM4_ESM.pdf]

California State University, Dominguez Hills
Study Information Sheet

Faculty/Students' Perspectives, Knowledge, Attitudes, and Practices of Artificial Intelligence (AI) in Health Sciences and Nursing Education

Introduction:

You are being asked to participate in a research study conducted by Dr Obinna Oleribe (DrPH, MBA, MBBS), Dr Matt Mutchler (Ph.D.), and Dr Parichart Sabado, (PhD, MPH), from the School of Public Health and Health Sciences, College of Health, Human Services and Nursing at California State University, Dominguez Hills. As a faculty member/student over 18 years of age in the College of Health, Human Services, and Nursing, you are invited to participate in this study.

Purpose and Description of the Study:

The purpose of this study is to document the perspectives, knowledge, attitudes, and practices of faculty/students in the College of Health, Human Services, and Nursing to artificial intelligence (AI). If you decide to participate in this study, you will complete a self-administered questionnaire (survey). The survey has questions that explore your knowledge, understanding, and use of AI. We will also collect some demographic information, but no personally identifiable data will be collected. The survey should take approximately 15 minutes to complete.

Risk(s) and Discomfort(s):

There are no risk(s) associated with this study. Participation in this study is voluntary and if you volunteer to participate in this study, you may withdraw at any time without any consequences. You may also refuse to answer any questions you don't want to answer and still remain in the study.

Benefits:

By participating in this study, you will contribute to science, advance AI evolution, and provide vital information for evidence-based decisions.

Confidentiality:

The study is anonymous, so any information provided cannot be traced back to you. Please do not include your name or other identifying information in your survey responses that can identify you. Data will be analyzed in aggregate and stored in a secure location outside the reach of non-investigators.

Contact information:

If you have any questions or concerns about the research, please feel free to contact Obinna Oleribe at ooleribe@csudh.edu. If you have questions regarding your rights as a research participant, contact the California State University, Dominguez Hills IRB Office at 310-243-3756 or irb@csudh.edu.

This study information sheet is for you to keep. Please save or print a copy of this page for your records or take a screenshot of it. If you agree to participate, please Check YES to begin the survey.

☐ **YES** ☐ **NO**
